# Noninvasive Gamma Sensory Stimulation May Reduce White Matter and Myelin Loss in Alzheimer’s Disease

**DOI:** 10.3233/JAD-230506

**Published:** 2024-01-02

**Authors:** Xiao Da, Evan Hempel, Yangming Ou, Olivia Elizabeth Rowe, Zach Malchano, Mihály Hajós, Ralph Kern, Jonathan Thomas Megerian, Aylin Cimenser

**Affiliations:** a Cognito Therapeutics, Inc., Cambridge, MA, USA; b Department of Radiology, Harvard Medical School, Boston, MA, USA; c Department of Comparative Medicine, Yale University School of Medicine, New Haven, CT, USA

**Keywords:** Alzheimer’s disease, dementia, myelin, white matter

## Abstract

**Background:**

Patients with Alzheimer’s disease (AD) demonstrate progressive white matter atrophy and myelin loss. Restoring myelin content or preventing demyelination has been suggested as a therapeutic approach for AD.

**Objective:**

Herein, we investigate the effects of non-invasive, combined visual and auditory gamma-sensory stimulation on white matter atrophy and myelin content loss in patients with AD.

**Methods:**

In this study, we used the magnetic resonance imaging (MRI) data from the OVERTURE study (NCT03556280), a randomized, controlled, clinical trial in which active treatment participants received daily, non-invasive, combined visual and auditory, 40 Hz stimulation for six months. A subset of OVERTURE participants who meet the inclusion criteria for detailed white matter (N = 38) and myelin content (N = 36) assessments are included in the analysis. White matter volume assessments were performed using T1-weighted MRI, and myelin content assessments were performed using T1-weighted/T2-weighted MRI. Treatment effects on white matter atrophy and myelin content loss were assessed.

**Results:**

Combined visual and auditory gamma-sensory stimulation treatment is associated with reduced total and regional white matter atrophy and myelin content loss in active treatment participants compared to sham treatment participants. Across white matter structures evaluated, the most significant changes were observed in the entorhinal region.

**Conclusions:**

The study results suggest that combined visual and auditory gamma-sensory stimulation may modulate neuronal network function in AD in part by reducing white matter atrophy and myelin content loss. Furthermore, the entorhinal region MRI outcomes may have significant implications for early disease intervention, considering the crucial afferent connections to the hippocampus and entorhinal cortex.

## INTRODUCTION

The multifactorial [1] etiology of Alzheimer’s disease (AD) is often associated with several comorbidities [2], thus complicating the identification of effective treatment strategies. Although AD is characterized by the presence of amyloid-β (Aβ) containing extracellular plaques and tau-containing intracellular neurofibrillary tangles, decreased synaptic density is an early feature of AD [3]. Recent studies expanded the concept of brain plasticity to include activity-dependent changes in white matter structure and organization [4, 5] and suggested restoring myelin or preventing myelin loss as one treatment strategy [6]. White matter degeneration can affect brain function and connectivity, as myelin ensheathes axons and facilitates local and distant communication. Due to the crucial role of myelin in electrical impulse conduction within neuronal networks, longitudinal evaluation of white matter volume and myelin content could provide valuable insight into AD progression.

Previous studies have evaluated myelin content through routinely collected magnetic resonance imaging (MRI) to facilitate clinical implementation, especially when access to advanced quantitative MRI techniques may not be readily available. This approach utilizes T1-weighted (T1w) and T2-weighted (T2w) MRI acquisition sequences. T1w sequences are used to study anatomical structures of the brain. Due to the short relaxation time, T1w sequences are characterized by better contrast-to-noise ratio in white matter. The intensities of white matter in T1w sequences are provided by the spatial distribution of myelin-bound cholesterol, which contributes the most to the contrast in T1w images of the brain. On the other hand, given that T2 relaxation time is associated with proton transfers, molecular exchange, and diffusion of water, T2w sequences can be applied to better differentiate structural differences in regions with high water content, which is important for many disease process diagnoses. Since molecular motion of protons is constrained by hydrophobic properties of the lipid bilayer in myelin, relatively larger myelin content results in relatively lower intensity on T2w images. Therefore, T1w/T2w ratio images can provide a proxy estimate of myelin content and are used as a practical option to study myelin content.

Previous studies have demonstrated positive effects of 40 Hz gamma-sensory stimulation in AD transgenic mouse models [7–10] and in patients across the AD spectrum [11–13]. However, the potential impact of the gamma stimulation therapy on white matter volume and myelin content has not been studied. Of special interest are white matter changes in the entorhinal region, which contain bidirectional connections that support information transfer between cortical and hippocampal networks impacted in the early stages of AD, as shown in histological and neuroimaging studies [14].

Herein, we describe the effect of combined visual and auditory 40 Hz gamma-sensory stimulation on changes in white matter volume and myelin content in patients with mild cognitive impairment (MCI) or mild-moderate AD using volumetric MRI (T1w) and T1w/T2w ratios to evaluate white matter atrophy and regional differences in myelin content, respectively. We hypothesize that daily 1-h combined visual and auditory 40 Hz gamma-sensory stimulation therapy for a 6-month period may prevent oligodendrocyte damage or modify other pathological processes that may lead to a reduction in myelin loss, protect axons, and attenuate white matter atrophy in patients with MCI or mild-moderate AD.

## MATERIALS AND METHODS

### Study population and design

This analysis is based on data from the OVERTURE, Phase I/II randomized, placebo-controlled clinical trial (NCT03556280), https://clinicaltrials.gov/ct2/show/NCT03556280. The study evaluated the safety, tolerability, adherence, and efficacy of combined visual and auditory gamma-sensory stimulation treatment in participants with MCI and mild-moderate AD (Mini-Mental State Examination (MMSE) scores 14–26). The study was reviewed and approved by Advarra IRB (FDA IORG#0000635, OHRA IRB Registration #00000971). Informed consent was obtained from all participants. In cases where subjects were not competent to provide informed consent, a Legally Authorized Representative provided it and the individual participant assented to the research. At screening, participants with confounding pathology, such as ischemic stroke, intracerebral macro-hemorrhages, or more than four micro-hemorrhages, were excluded. Exclusion criteria also included profound hearing or visual impairments, seizure history, and the use of anti-seizure/anti-epileptic medication. During the trial, cholinesterase inhibitors were permitted at a stable dose. Memantine was not permitted. During OVERTURE, 46 participants were assigned to active treatment, of which 33 completed the study, and 28 participants were assigned to sham treatment, of which 20 completed the study. Active treatment group participants received daily, 1-hour 40 Hz simultaneous audio-visual sensory stimulation for a 6-month period while sham group participants received sham stimulation for the same period. For each participant, the volume of the auditory stimulation and the intensity of the visual stimulation were set within a range that was comfortable.

Analyses were conducted on all OVERTURE participants who met none of the exclusion criteria: The exclusion criteria for white matter volumetric analyses were as follows: (a) participants who declined > 4 standard deviations from the mean in multiple efficacy measures (1 sham participant excluded); (b) participants who did not have both baseline and end of study (i.e., Month 6) data for structural MRI (22 participants excluded, of which 21 of them did not complete the trial); (c) participants who did not have a sufficient T1w image quality, including excessive motion artifact and insufficient gray matter-white matter contrast (13 participants excluded). Overall, thirty-eight participants (25 Treatment and 13 Sham) were included in white matter volumetric assessments. Of the thirty-eight participants, two failed T2w image quality due to reconstruction error, and a total of thirty-six participants (24 Treatment and 12 Sham) were included for longitudinal T1w/T2w white matter myelin content assessments.

### Therapy device

The device used in this study was a gamma-sensory stimulation device (Figure 1B of [13]) developed by Cognito Therapeutics, Inc. It consisted of a handheld controller, an eye-set for visual stimulation, and headphones for auditory stimulation. During the therapy, participants could adjust the brightness of the visual stimulation and the volume of the auditory stimulation using push buttons on the controller. If assistance is needed, they could communicate with a care partner. The device captured usage information and adherence data. All the information was uploaded to a secured cloud server for remote monitoring.

### MRI data acquisition

Structural MRI was acquired at Baseline, Month 3 and Month 6 using 1.5 Tesla MRI scanners. The study adopted an ADNI1 comparable standardized MRI scan protocol. For T1w, it included 1.25×1.25 mm in-plane spatial resolution, 1.2 mm thickness, TR 2400 ms and TE 3.65 ms for Siemens Espree scanner, 0.94×0.94 mm in-plane spatial resolution, 1.2-mm thickness, TR ∼3.9 ms and TE 1.35 ms for General Electric scanner Signa HDxt and 0.94×0.94 mm in-plane spatial resolution, 1.2-mm thickness, TR 9.5 ms and TE ∼3.6 or 4 ms for Philips Ingenia scanner or Philips Achieva scanner. For T2w, it included 1×1 mm in-plane spatial resolution, 4 mm thickness, TR 3000 ms and TE 96 ms for Siemens and GE scanners and 1×1 mm in-plane spatial resolution, 4 mm thickness, TR 3000 ms and TE 92 ms for Philips scanner [15].

### Parcellation

FreeSurfer’s standard reconstructive pipeline (“recon-all”, version 7.2.0) was used to process and automatically parcellate T1 MRI data into 68 predefined, left and right hemispheres separated, white matter structures [16–23]. Per hemisphere white matter structures were joined to obtain 34 white matter structures that combined the two hemispheres. FreeSurfer was also used to automatically generate 12, left and right hemispheres separated, lobar white matter structures. Similarly, per hemisphere lobar white matter structures were also joined to obtain 6 lobar white matter structures that combined the two hemispheres. We focus on these 52, 12 hemispherical-based and 34 + 6 joined, white matter structures to assess changes in volume and myelin.

### Myelin-reflecting contrast

To acquire a myelin-reflecting contrast, a non-invasive imaging sensitive to the estimation of myelin content was employed by using T1w/T2w ratio [24–26]. This process included co-registration of the T2w images to the T1w images using rigid transformation, inhomogeneity correction for both T1w and T2w images and linear calibration of image intensity using non-brain tissue masks to create T1w/T2w ratio images corresponding to myelin content [27, 28]. T1w/T2w ratios were processed using MRTool (v. 1.4.3, https://www.nitrc.org/projects/mrtool/), the toolbox implemented in the SPM12 software (University College London, London, UK, http://www.fil.ion.ucl.ac.uk/spm).

### Statistical methods

Demographic and biomarker data of the active treatment group participants and the sham group participants were compared using two-sample *t*-tests for numerical data or chi-square tests for categorical data. To evaluate the efficacy, we calculated the change in volumetric data and myelin content in each of the white matter structures using the formula Change = 100*(*V*_*Follow*-*up*_/*V*_*Baseline*_ - 1) % where V is the volume or the myelin content. The changes were then assessed using a Bayesian linear mixed effects model. Non-informative priors are used for all effects of the model that includes total intracranial volume, baseline MMSE score, baseline age, visit (as number of days from the start of the treatment), group, baseline MRI measures (volume for white matter atrophy assessment and sum of the T1w/T2w ratios across each studied white matter structure for myelin content assessment), group-visit interaction and baseline MRI measure-visit interaction. Random effects of the model include subject and site information. The Kenward-Roger approximation of the degrees of freedom was used. For volumetric analysis, volume change (% change from baseline) and for myelination analysis, sum of T1w/T2w ratio change (% change from baseline) were assessed for each studied white matter structure. All statistical analyses were conducted using R (version 4.1.1).

## RESULTS

Thirty-eight participants (25 Active Treatment, 13 Sham) who completed the 6-month study and whose MRI met the study criteria were evaluated in the volumetric analysis, whereas thirty-six (24 Active Treatment, 12 Sham) were who met study criteria evaluated in the T1w/T2w myelin content analysis (T1w and T2w MRI images of a sample active treatment participant and a sham participant can be found in Supplementary Figure 1). At baseline, the sham treated group were older than the active treatment group (76.62±9.97 versus 68.36±7.69, *p* = 0.02), otherwise there were no significant differences in sex, MMSE, Alzheimer’s Disease Cooperative Study - Activities of Daily Living, *APOE4* status, white matter volume or T1w/T2w between the two groups (Table 1). A baseline comparison of Fazekas scores was conducted for the population where T1w/T2w myelin analysis is performed: The active group had 1 participant with grade 0, 18 participants with grade 1, and 5 participants with grade 2. The sham group had 8 participants with grade 1, 3 participants with grade 2, and 1 participant with grade 3. There was no statistically significant difference between the two groups. In the participants receiving active treatment, headaches, and tinnitus were the most reported adverse events. There were no observations of ARIA-E (vasogenic edema and sulcal effusions) or ARIA-H (hemosiderin deposit).

**Table 1:** Demographic, clinical, and white matter characteristics of the active treatment and the sham group participants at baseline

	Active Treatment (*n* = 25)	Sham (*n* = 13)	*p*
Age, y, mean±SD	68.36±7.69	76.62±9.97	0.02
Sex, Male/Female	7/18	8/5	0.10
MMSE^†^ score, mean±SD	20.64±3.15	19.77±3.27	0.44
ADCS-ADL^‡^ scale, mean±SD	64.88±7.95	66.23±10.83	0.70
Number (%) of *APOE*^¶^ *ɛ*4 positive	13 (52.00%)	7 (53.85%)	1
White matter volume^⊤^, mean±SD	449.78±71.57	459.07±57.07	0.66
T1w/T2w^§^, mean±SD	1.34±.23	1.29±0.30	0.61

^†^MMSE, Mini-Mental State Exam; ^‡^ADCS-ADL, Alzheimer’s Disease Cooperative Study - Activities of Daily Living; ^¶^*APOE*, apolipoprotein E. ^⊤^Unit: cm^3^. ^§^Active Treatment (*n* = 24), Sham (*n* = 12).

**Fig. 1:**
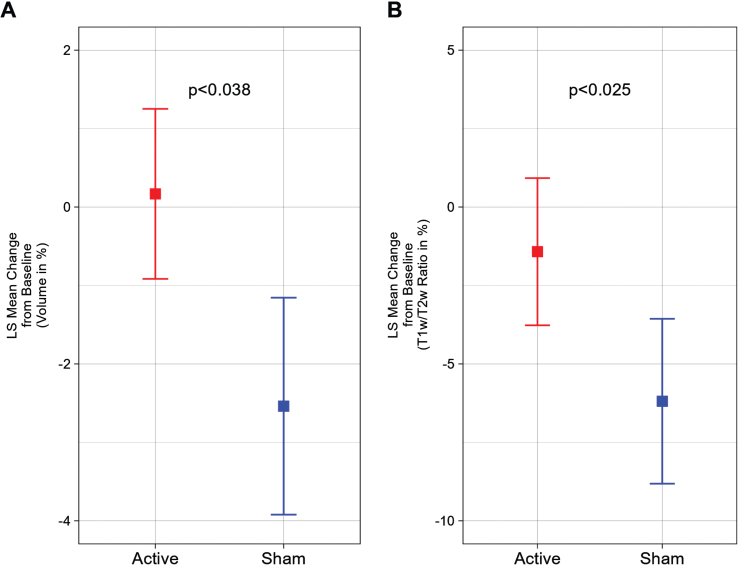
White matter volume and T1w/T2w ratio change from baseline (%) for a 6-month period; the statistically significant difference favors the active treatment group. A) Least Squares (LS) Mean volume changes for the total cerebral white matter shows the significant difference (*p* < 0.038) between the Active Treatment group participants (Red) and the Sham group participants (Blue), favoring the Active Treatment group. Error bars indicate SE. B) LS Mean sum of T1w/T2w ratio changes for the total cerebral white matter shows the significant difference (*p* < 0.025) between the Active Treatment group participants (Red) and the Sham group participants (Blue), favoring the Active Treatment group. Error bars indicate SE.

Compared to baseline MRI values, we observed that the active treatment group demonstrated a 0.17±1.08% (1.06±5.35 cm^3^) increase and the sham group demonstrated a –2.54±1.38% (–12.37±6.81 cm^3^) decrease in total cerebral white matter volume after a 6-month period, representing a statistically significant difference (*p* < 0.038) (Fig. 1A). Furthermore, a statistically significant (*p* < 0.025) difference was also observed in the myelin-reflecting T1w/T2w ratio between groups; the active treatment group demonstrated a –1.42±2.35% decrease and the sham group demonstrated a –6.19±2.63% decrease (Fig. 1B).

**Fig. 2:**
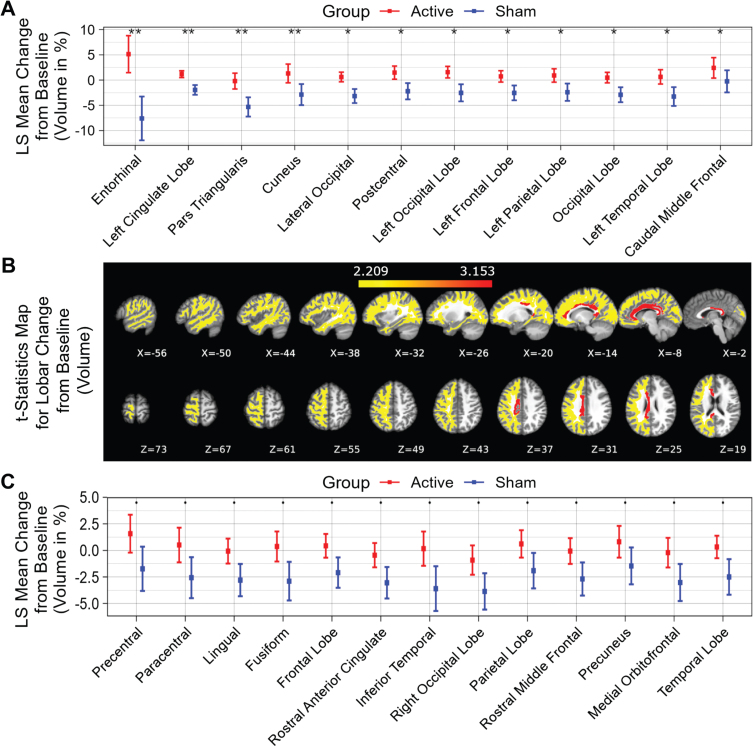
White matter structures volume change from baseline (%) for a 6-month period; all statistically significant differences favor the active treatment group. LS Mean volume changes for the white matter structures (A), sorted in ascending order by *p*-value, shows the significant difference (*p* < 0.05) between the Active Treatment group participants (Red) and the Sham group participants (Blue), favoring the active treatment group. (B), Maps of the t-statistics showing LS Mean lobar white matter volume changes of the difference between the active treatment group and sham group. The colorbar depicts the values of the T-maps after thresholding at the *p* = 0.05 level; a high value indicates more lobar white matter volume preservation in the active treatment group. The images are shown in neurological convention. LS Mean volume changes for the white matter structures (C), sorted in ascending order by *p* value, shows the marginal difference (0.05≤*p* < 0.1) between the Active Treatment group participants (Red) and the Sham group participants (Blue), favoring the Active Treatment group. Error bars indicate SE. ** for *p* < 0.01, * for 0.01≤*p* < 0.05, and • for 0.05 ≤ *p* < 0.1.

Fifty-two distinct white matter structures were analyzed based on volume (Supplementary Table 1) and myelin-reflecting T1w/T2w ratio (Supplementary Table 2) changes from baseline following a 6-month daily gamma visual and auditory sensory stimulation. We observed that all statistically significant changes favored the active treatment group: Compared to the sham group, significant (*p* < 0.05) attenuation in volume loss was found in 12 of 52 structures: entorhinal region, left cingulate lobe, pars triangularis region, cuneus region, lateral occipital region, postcentral region, left occipital lobe, left frontal lobe, left parietal lobe, occipital lobe, left temporal lobe and caudal middle frontal region (sorted in ascending order by *p* value) for the active treatment group after 6 months of treatment (Fig. 2A, see Fig. 2B for maps of T-statistics depicting the differences between the two groups in lobar white matter volume). Forty Hz gamma-sensory stimulation therapy for a 6-month period prevented white matter atrophy in the entorhinal region: The active treatment group demonstrated a 5.14±3.66% (0.08±0.06 cm^3^) increase, while the sham group demonstrated a –7.60±4.35% (–0.13±0.07 cm^3^) decrease in volume. The difference between these two groups was statistically significant (*p* < 0.002). The treatment also trended in the direction of preventing volume loss (0.05≤*p* < 0.1) in the precentral region, paracentral region, lingual region, fusiform region, frontal lobe, rostral anterior cingulate region, inferior temporal region, right occipital lobe, parietal lobe, rostral middle frontal, precuneus region, medial orbitofrontal region, and temporal lobe (sorted in ascending order by *p* value) (Fig. 2C). The full extended results of volume changes for 52 white matter structures from baseline for a 6-month period are shown in Table 2.

**Table 2:** **LS Mean volume changes for 52 white matter structures from baseline (%) for a 6-month period**. All the statistically significant changes favor the Active Treatment group over the Sham group. Light orange shaded areas for *p* < 0.01, light green shaded areas for 0.01≤*p* < 0.05 and light-yellow shaded areas for 0.05≤*p* < 0.1

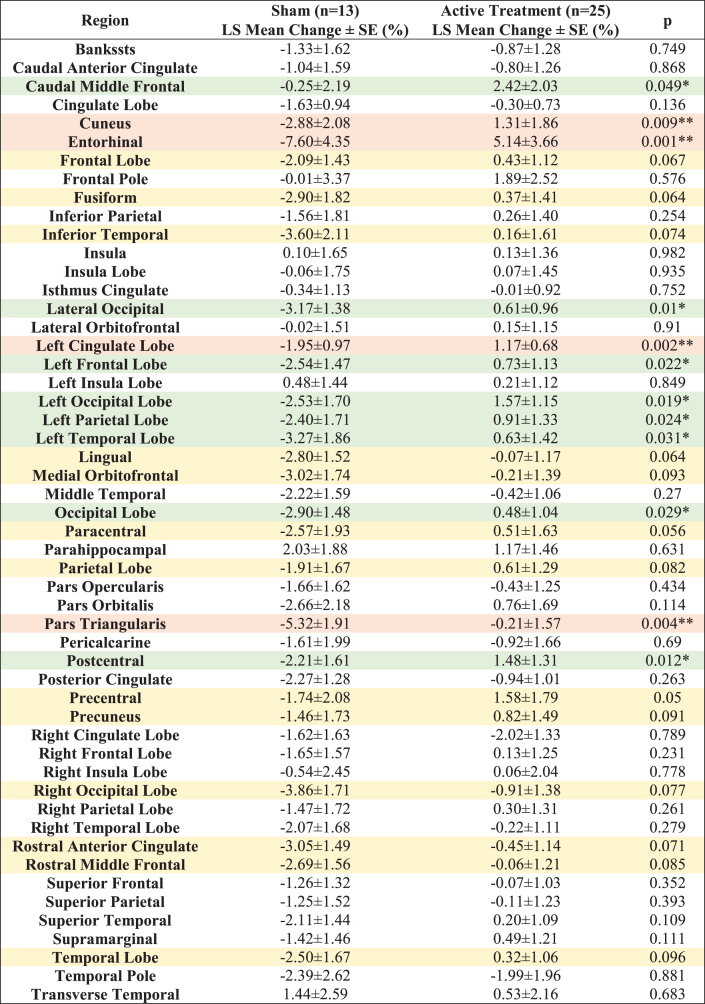

**Fig. 3:**
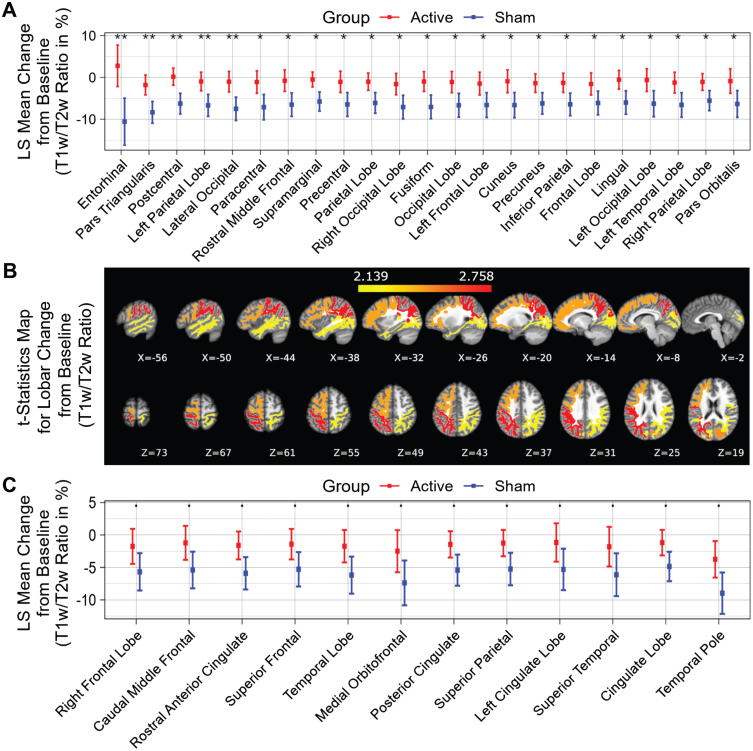
T1w/T2w ratio change in white matter structures (% change from baseline) for a 6-month period; all statistically significant differences favor the active treatment group. LS Mean sum of T1w/T2w ratio changes in the white matter structures (A), sorted in ascending order by *p*-value, shows the significant difference (*p* < 0.05) between the Active Treatment group participants (Red) and the Sham group participants (Blue), favoring the active treatment group. (B), Maps of the t-statistics showing LS Mean lobar white matter myelin content changes of the difference between the active treatment group and sham group. The colorbar depicts the values of the T-maps after thresholding at the *p* = 0.05 level; a high value indicates more lobar white matter myelin preservation in the active treatment group. The images are shown in neurological convention. LS Mean sum of T1w/T2w ratio changes in the white matter structures (C), sorted in ascending order by *p*-value, shows the marginal difference (0.05≤*p* < 0.1) between the Active Treatment group participants (Red) and the Sham group participants (Blue), favoring the Active Treatment group. Error bars indicate SE. ** for *p* < 0.01, * for 0.01≤*p* < 0.05, and • for 0.05 ≤ *p* < 0.1.

Compared to the sham group, significantly less myelin content loss (smaller T1w/T2w ratio change) was observed in the entorhinal region, pars triangularis region, postcentral region, left parietal lobe, lateral occipital region, paracentral region, rostral middle frontal region, supramarginal region, precentral region, parietal lobe, right occipital lobe, fusiform region, occipital lobe, left frontal lobe, cuneus region, precuneus region, inferior parietal region, frontal lobe, lingual region, left occipital lobe, left temporal lobe, right parietal lobe and pars orbitalis region (Fig. 3A, white matter structures sorted in ascending order by *p* value), indicating significant differences (*p* < 0.05) between the active treatment group and the sham group (see Fig. 3B for maps of T-statistics depicting the differences between the two groups in lobar white matter myelin content). Within the 52 studied white matter structures, the most significant myelin content T1w/T2w ratio change was also in the entorhinal region. The active treatment group participants exhibit a 2.78±4.97% increase from baseline on T1w/T2w ratio while the sham group participants exhibit a –10.59±5.63% decrease from baseline on sum of T1w/T2w ratio (*p* < 0.003), suggesting that 40 Hz gamma-sensory stimulation therapy for a 6-month period may significantly protect myelination. The treatment may also trend towards slowing down demyelination (0.05≤*p* < 0.1) in the right frontal lobe, caudal middle frontal region, rostral anterior cingulate region, superior frontal region, temporal lobe, medial orbitofrontal region, posterior cingulate region, superior parietal region, left cingulate lobe, superior temporal region, cingulate lobe, and temporal pole region (Fig. 3C, white matter structures sorted in ascending order by *p* value). All myelin content T1w/T2w ratio changes in the 52 white matter structures from baseline for a 6-month period are shown in Table 3.

**Table 3:** **LS Mean T1w/T2w ratio changes in 52 white matter structures from baseline (%) for a 6-month period**. All the statistically significant changes favor the Active Treatment group over the Sham group. Light orange shaded areas for *p* < 0.01, light green shaded areas for 0.01≤*p* < 0.05 and light-yellow shaded areas for 0.05≤*p* < 0.1

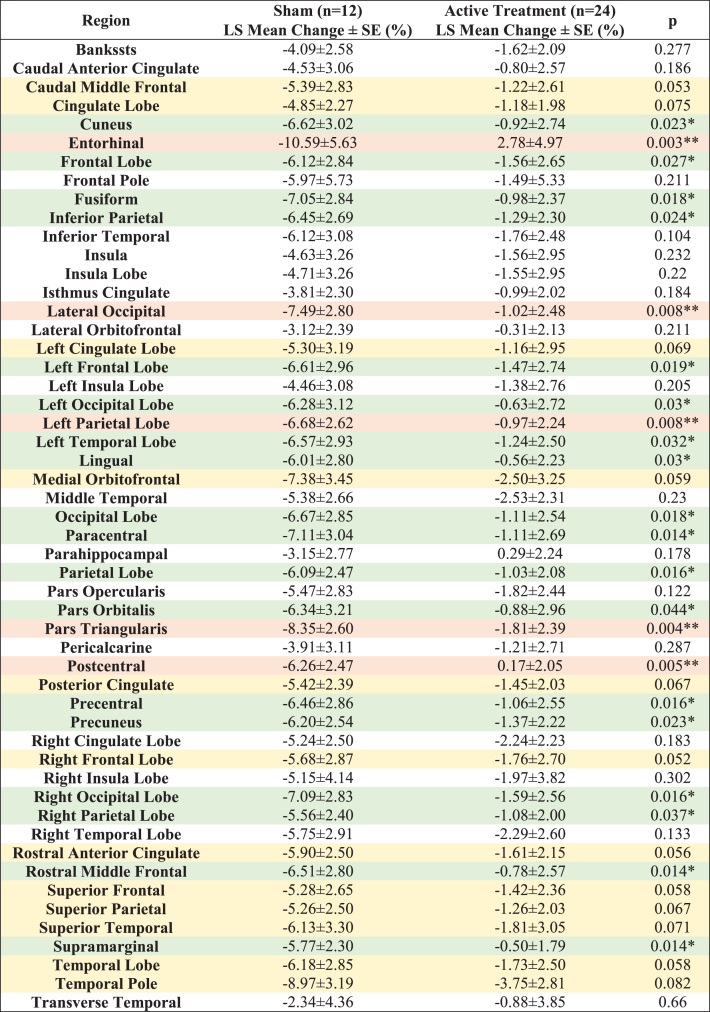

While the sham-treated group was older at baseline and age was included as a covariate in our models, it did not contribute significantly to any of the changes we observed over the course of six months.

## DISCUSSION

Our data suggests that daily, 1-hour 40 Hz combined visual and auditory gamma-sensory stimulation therapy for a 6-month period in participants with AD resulted in reduced white matter atrophy and myelin content loss compared to sham treatment. We also consistently observed reduced myelin content loss (T1w/T2w ratio) across the brain same regions. Overall, the effect of gamma-sensory stimulation on decreasing white matter atrophy and myelin loss was greatest in the entorhinal region. Although we observed a statistically significant difference in change of white matter volume and myelin content between active and sham arm participants after six months of treatment, and a positive change in some regions in the active group, owing to the small sample size, it is unclear whether 40 Hz gamma sensory stimulation increases white matter volume or simply prevents atrophy.

While the extracellular accumulation of amyloid plaques and intra-neuronal presence of tau-containing neurofibrillary tangles may lead to neuron and synapse loss in AD, there is evidence that myelin damage in preclinical AD independently contributes to disease progression [29, 30]. Studies have revealed that white matter degeneration and myelin damage may be associated with neuronal dysfunction and cognitive decline [31–33].

Subsequent studies suggest that white matter atrophy and myelin loss may be a mechanistically important AD treatment target [34] and may identify individuals at high risk of disease progression. Based on experimental results, myelin preservation has recently been suggested as a therapeutic strategy for improving cognition in AD [6]. Furthermore, AD amyloid pathology may be affected by myelin alterations, which precede the onset of amyloid and tau pathological changes [29, 35]. The potential link between white matter degeneration and clinical disease progression [34] provides an important perspective to view results from gamma-sensory stimulation therapy as a potential disease modifying approach to slow AD progression.

Preservation of entorhinal white matter by this innovative treatment may be particularly relevant to AD given its afferent connections into the hippocampus and the entorhinal cortex [14, 36]. Due to densely concentrated axons of the perforant path in the white matter entorhinal region, stimulation of white matter in the entorhinal region recruits many of these axons and benefits subsequent memory during learning [36, 37]. Preservation of white matter volume and myelin content by 40 Hz combined visual and auditory gamma-sensory stimulation may help protect the existing connections and prevent further damage to this region. The white matter lobar regions, particularly in the left hemisphere, that showed statistically significant differences between active and sham treatment in our analysis, are known to be affected in AD. Studies have demonstrated that damage to the temporal lobe affects memory and that temporal lobe predominant damage, specifically atrophy of the medial temporal lobe, is the most predictive structural brain biomarker for AD [38, 39]. Early-onset AD patients exhibit bilateral posterior myelin loss spreading to the temporal areas of their left temporal area, while late-onset AD patients exhibit distributed bilateral myelin loss affecting the temporal and cingulate areas [40, 41]. A separate diffusion MRI study additionally demonstrated the directional diffusivity changes with a decrease in axial diffusivity and an increase in radial diffusivity in temporal white matter of AD patients, suggesting entire myelinated axonal loss as observed in Wallerian degeneration [42]. Severe white matter occipital atrophy has been observed in the posterior cortical atrophy variant of early age-of-onset AD [43], and there is evidence that neuropathological abnormalities of the occipital lobe may lead to visual hallucinations in AD [44]. Although myelin sheath structural integrity deteriorates with normal aging, particularly in regions of late myelination like the frontal lobe, it has been shown that this degradation is more severe in AD patients [45]. One AD development model that aims to characterize the chain of pathological events leading to AD pathology and diagnosis highlights the importance of the parietal lobe [38]. In this model, amyloid accumulation crosses a threshold because of myelin breakdown and disconnection between the posterior cingulate gyrus/precuneus and the medial temporal lobe and leads to a cascade of events.

Given the practical clinical implementation and the direct correspondence to regional differences in myelin, myelin-reflecting T1w/T2w ratio has been applied to non-invasively study white matter pathology in other neurological disorders. In schizophrenia, the regions identified in white matter by using T1w/T2w ratio are consistent with previous studies linking cerebellar deficits to neurological signs [28]. Based on their findings, the authors concluded that T1w/T2w ratio can yield improved differentiation from healthy controls than studying T1w and T2w images alone. In multiple sclerosis, the T1w/T2w ratio has been used to characterize microstructural changes in myelin and neuroaxonal integrity [46]. T1w/T2w ratios are lower in lesioned white matter regions compared to non-lesioned white matter regions, consistent with known disease-related reduced myelin content, while no differences have been observed in the T1w/T2w ratio in normal-appearing white matter regions of multiple sclerosis subjects and the white matter of controls. Nonetheless, another study found that patients with clinically isolated syndrome, some of whom later developed multiple sclerosis, a decrease in T1w/T2w was demonstrated prior to the onset of lesion formation [47].

The specific mechanisms that contribute to preservation of white matter and myelin by 40 Hz combined visual and auditory sensory stimulation is unclear, although synaptic and non-synaptic effects on oligodendrocytes (OL) may ultimately lead to positive effects within myelin structures. For example, non-invasive gamma stimulation may lead to increased axon-glia signaling at functional synapses between neurons and oligodendrocyte precursor cells (OPC) and hence influence OPC [48]. In addition, release of neurotransmitter-filled vesicles of neurons at non-synaptic junctions with OPC may promote myelination. These hypotheses are consistent with *in-vivo* studies where proliferation of OPC and their development into OL, extension and stabilization of myelin sheaths, regulation of myelinating capacity of OL are achieved by changing the neuronal activity via external stimulation or by placing animals into an enriched environment [49].

The relatively new understanding of white matter as a non-static, dynamic, adaptive structure, extending into adulthood has led to increased interest in examining glial contributions to disease onset and progression. Stimulation-induced neuronal activity can change white matter properties, i.e., white matter plasticity; which can lead to changes in myelin density, affecting the speed, precision, and timing of axonal signal conduction, leading to optimal synchronization of spike-time arrival which plays a crucial role in optimizing neuronal network function. The regulation of myelination and adaptive myelination plays an important role in the temporal structure of neural interactions as a means to achieve self-organization and influence the dynamics of the network function. It also shifts the focus from a synaptic strength only model to one that also incorporates the role of glial-mediated self-organizing networks, which can reorganize the timing of neural interactions to preserve specific network target dynamics and achieves brain homeostasis [5].

Understanding that white matter properties may be modified, and myelin may be regulated in an activity dependent manner may advance our understanding of white matter plasticity and its role in achieving optimal network dynamics. Our results demonstrate the positive effects of combined visual and auditory gamma-sensory stimulation on white matter atrophy and myelin content. This may ultimately contribute to restoring neural network function in AD and in other neurodegenerative disorders.

To the best of our knowledge, the T1w/T2w ratio in white matter has not been previously studied in AD patients. Despite its merits, there are limitations to consider. The first is that MRI acquired with very different pulse sequences at different imaging centers may generate variations in image contrast. The originally introduced MRI scan protocol [26] used isotropic voxels for both T1w and T2w images. In our MRI scan protocol, like ADNI1 protocol, the voxels of T2w images were not isotropic. Secondly, in addition to myelin alteration, other pathological changes such as edema, inflammation, iron accumulation, free water or fiber density may also change T1w/T2w ratio in individuals with central nervous system disorders [28, 50]. It is critically important for future studies to consider confounding effects including iron and inflammation and assess T1w/T2w ratio in both healthy and pathological tissue histologically. Furthermore, it is important for future studies to add other advanced MRI imaging modalities such as diffusion tensor imaging, neurite orientation and dispersion density imaging, quantitative susceptibility mapping, multicomponent driven equilibrium single pulse observation of T1 and T2 (mcDEPOT), and magnetization transfer ratio imaging to improve both the sensitivity and the specificity of myelin content quantification.

Our research represents a retrospective evaluation of a Phase II study. Our foremost aim was to pinpoint specific endpoints, with the anticipation of their prospective utilization in future studies, underpinned by a more robust sample size. Our results suggest that 40 Hz combined visual and auditory gamma-sensory stimulation therapy may have beneficial effects on MRI pathophysiological features of AD and other neurodegenerative diseases. Six months of treatment significantly attenuated total and regional white matter volume loss and significantly reduced myelin content loss, consistently across the same brain regions. Specifically, white matter structures with statistically significant differences in volume or myelin content were better preserved in the active treatment group with the most significant difference in the entorhinal region, which is an important structure relevant to AD pathology. (See a comparison of *p* values and adjusted *p* values for white matter volume (Supplementary Table 3) and myelin content (Supplementary Table 4). Further larger studies (such as the HOPE pivotal study (NCT05637801)) may provide additional insights into this promising therapeutic strategy to improve function and cognition in AD and possibly other neurodegenerative diseases that are vulnerable to white matter abnormalities.

## Supplementary Material

Supplementary Material

## Data Availability

The data supporting the findings of this study are available on request from the corresponding author. The data are not publicly available due to privacy or ethical restrictions.
