# Supplementary material for: Noninvasive Gamma Sensory Stimulation May Reduce White Matter and Myelin Loss in Alzheimer’s Disease
Source: J Alzheimers Dis. 2024 Jan 2;97(1):359–72. doi: 10.3233/JAD-230506 (PMC10789351; doi:10.3233/JAD-230506)
Supplement: Supplementary Material [file jad-97-jad230506-s001.pdf]

Supplementary Material

Noninvasive Gamma Sensory Stimulation May Reduce White Matter and Myelin Loss in Alzheimer's Disease

Supplementary Table 1. White matter volume (cm^3) for 52 structures at baseline. No statistically significant difference between the Sham group and the Active Treatment group was observed for white matter volumes for all 52 white matter structures at baseline.

Region	Sham (n=13) Mean ± SE	Active Treatment (n=25) Mean Change ± SE	p
**Bankssts**	5.21±0.20	5.27±0.21	0.817
**Caudal Anterior Cingulate**	4.67±0.22	4.76±0.15	0.740
**Caudal Middle Frontal**	12.64±0.53	11.97±0.40	0.328
**Cingulate Lobe**	24.03±0.94	23.78±0.61	0.825
**Cuneus**	6.40±0.29	5.75±0.23	0.091
**Entorhinal**	1.73±0.12	1.70±0.09	0.883
**Frontal Lobe**	149.04±5.26	145.67±4.45	0.629
**Frontal Pole**	0.75±0.04	0.69±0.02	0.218
**Fusiform**	12.68±0.38	12.48±0.42	0.729
**Inferior Parietal**	20.72±0.88	20.62±0.79	0.930
**Inferior Temporal**	11.87±0.38	12.14±0.53	0.677
**Insula**	18.71±0.73	18.29±0.61	0.669
**Insula Lobe**	18.47±0.75	18.18±0.60	0.771
**Isthmus Cingulate**	7.40±0.27	7.02±0.22	0.279
**Lateral Occipital**	22.24±0.65	20.64±0.72	0.108
**Lateral Orbitofrontal**	14.08±0.53	13.37±0.38	0.293
**Left Cingulate Lobe**	12.65±0.57	12.48±0.33	0.798
**Left Frontal Lobe**	74.08±2.83	72.59±2.27	0.684
**Left Insula Lobe**	9.37±0.37	9.19±0.31	0.702
**Left Occipital Lobe**	23.23±0.61	22.00±0.80	0.231
**Left Parietal Lobe**	48.20±1.91	46.96±1.73	0.636
**Left Temporal Lobe**	31.81±1.16	31.40±1.18	0.805
**Lingual**	12.17±0.51	11.61±0.36	0.381
**Medial Orbitofrontal**	8.39±0.27	7.91±0.25	0.199
**Middle Temporal**	11.22±0.40	10.90±0.45	0.596
**Occipital Lobe**	47.90±1.42	44.67±1.53	0.130
**Paracentral**	9.10±0.39	8.80±0.28	0.536
**Parahippocampal**	2.91±0.12	3.01±0.09	0.514
**Parietal Lobe**	97.84±3.60	96.07±3.31	0.720
**Pars Opercularis**	6.63±0.28	6.36±0.24	0.462
**Pars Orbitalis**	2.43±0.11	2.33±0.07	0.425
**Pars Triangularis**	6.49±0.26	6.09±0.21	0.242
**Pericalcarine**	7.81±0.30	7.12±0.32	0.120
**Postcentral**	16.50±0.82	15.77±0.52	0.460
**Posterior Cingulate**	7.88±0.31	7.97±0.19	0.797

**Precentral**	31.14±1.35	29.47±0.99	0.328
**Precuneus**	18.79±0.81	18.92±0.75	0.908
**Right Cingulate Lobe**	11.37±0.41	11.30±0.30	0.878
**Right Frontal Lobe**	74.96±2.50	73.09±2.20	0.578
**Right Insula Lobe**	9.10±0.41	9.00±0.32	0.852
**Right Occipital Lobe**	24.67±0.88	22.66±0.81	0.103
**Right Parietal Lobe**	49.65±1.75	49.11±1.63	0.823
**Right Temporal Lobe**	30.46±0.95	29.90±1.06	0.695
**Rostral Anterior Cingulate**	4.24±0.19	4.20±0.14	0.866
**Rostral Middle Frontal**	24.33±0.94	24.50±1.32	0.915
**Superior Frontal**	34.27±1.10	35.52±1.32	0.470
**Superior Parietal**	24.99±1.03	24.20±0.77	0.542
**Superior Temporal**	15.78±0.61	15.06±0.58	0.395
**Supramarginal**	18.46±0.76	17.99±0.70	0.651
**Temporal Lobe**	62.27±1.98	61.30±2.23	0.745
**Temporal Pole**	1.59±0.09	1.41±0.04	0.086
**Transverse Temporal**	1.60±0.08	1.56±0.07	0.644

Supplementary Table 2. T1w/T2w ratio for 52 structures at baseline. No statistically significant difference between the Sham group and the Active Treatment group was observed for T1w/T2w ratio in all 52 white matter structures at baseline.

Region	Sham (n=12) Mean ± SE	Active Treatment (n=24) Mean ± SE	p
**Bankssts**	1.25±0.08	1.33±0.07	0.408
**Caudal Anterior Cingulate**	1.11±0.10	1.19±0.05	0.460
**Caudal Middle Frontal**	1.28±0.10	1.27±0.05	0.951
**Cingulate Lobe**	1.27±0.09	1.32±0.05	0.651
**Cuneus**	1.37±0.12	1.34±0.06	0.860
**Entorhinal**	1.24±0.11	1.29±0.07	0.674
**Frontal Lobe**	1.33±0.09	1.36±0.05	0.760
**Frontal Pole**	2.31±0.14	2.34±0.08	0.856
**Fusiform**	1.20±0.08	1.27±0.05	0.494
**Inferior Parietal**	1.27±0.08	1.33±0.06	0.594
**Inferior Temporal**	1.17±0.06	1.25±0.05	0.296
**Insula**	1.70±0.12	1.73±0.06	0.813
**Insula Lobe**	1.69±0.12	1.72±0.06	0.798
**Isthmus Cingulate**	1.49±0.09	1.47±0.05	0.809
**Lateral Occipital**	1.19±0.08	1.20±0.04	0.936
**Lateral Orbitofrontal**	1.58±0.11	1.56±0.05	0.863
**Left Cingulate Lobe**	1.27±0.09	1.32±0.05	0.643
**Left Frontal Lobe**	1.32±0.09	1.35±0.05	0.761
**Left Insula Lobe**	1.71±0.12	1.74±0.06	0.811
**Left Occipital Lobe**	1.27±0.07	1.29±0.05	0.826
**Left Parietal Lobe**	1.23±0.09	1.28±0.05	0.628
**Left Temporal Lobe**	1.26±0.07	1.31±0.05	0.650
**Lingual**	1.35±0.08	1.41±0.06	0.538
**Medial Orbitofrontal**	1.59±0.09	1.59±0.05	0.974
**Middle Temporal**	1.26±0.07	1.28±0.05	0.849
**Occipital Lobe**	1.25±0.08	1.26±0.05	0.877
**Paracentral**	1.28±0.09	1.33±0.05	0.632
**Parahippocampal**	1.02±0.08	1.14±0.06	0.284
**Parietal Lobe**	1.27±0.09	1.32±0.05	0.587
**Pars Opercularis**	1.25±0.08	1.27±0.06	0.784
**Pars Orbitalis**	1.81±0.14	1.81±0.06	0.973
**Pars Triangularis**	1.48±0.09	1.44±0.04	0.676
**Pericalcarine**	1.19±0.07	1.19±0.06	0.980
**Postcentral**	1.29±0.11	1.32±0.05	0.836
**Posterior Cingulate**	1.24±0.09	1.34±0.05	0.370
**Precentral**	1.33±0.09	1.35±0.04	0.786
**Precuneus**	1.26±0.11	1.34±0.05	0.547
**Right Cingulate Lobe**	1.27±0.09	1.32±0.05	0.671
**Right Frontal Lobe**	1.34±0.09	1.37±0.05	0.761
**Right Insula Lobe**	1.67±0.12	1.70±0.06	0.794
**Right Occipital Lobe**	1.23±0.08	1.24±0.05	0.924

**Right Parietal Lobe**	1.31±0.09	1.37±0.05	0.554
**Right Temporal Lobe**	1.27±0.08	1.32±0.05	0.596
**Rostral Anterior Cingulate**	1.21±0.09	1.24±0.05	0.825
**Rostral Middle Frontal**	1.29±0.09	1.31±0.05	0.892
**Superior Frontal**	1.21±0.08	1.30±0.05	0.390
**Superior Parietal**	1.11±0.07	1.18±0.05	0.446
**Superior Temporal**	1.48±0.10	1.46±0.05	0.902
**Supramarginal**	1.52±0.10	1.56±0.06	0.727
**Temporal Lobe**	1.26±0.08	1.31±0.05	0.619
**Temporal Pole**	1.43±0.09	1.34±0.05	0.446
**Transverse Temporal**	1.59±0.11	1.61±0.07	0.871

Supplementary Table 3. Comparison of p values and adjusted p values of white matter volume changes from baseline for a 6-month period. Raw p values and adjusted p values, using Benjamini-Hochberg procedure (FDR), are shown. For FDR, an assumption of independence between subregions within a lobe is made. Two subregions that have p values less than 0.05 have FDR Corrected p values greater than 0.05. Light orange shaded areas for p<0.01, light green shaded areas for 0.01≤p<0.05, and light-yellow shaded areas for 0.05≤p<0.1.

Lobe	p	FDR Corrected p
**Frontal**		
Caudal Middle Frontal	0.049*	0.154
Frontal Pole	0.576	0.634
Lateral Orbitofrontal	0.91	0.91
Medial Orbitofrontal	0.093	0.17
Paracentral	0.056	0.154
Pars Opercularis	0.434	0.53
Pars Orbitalis	0.114	0.179
Pars Triangularis	0.004**	0.044*
Precentral	0.05	0.154
Rostral Middle Frontal	0.085	0.17
Superior Frontal	0.352	0.484
**Temporal**		
Bankssts	0.749	0.843
Entorhinal	0.001**	0.009**
Fusiform	0.064	0.222
Inferior Temporal	0.074	0.222
Middle Temporal	0.27	0.486
Parahippocampal	0.631	0.843
Superior Temporal	0.109	0.245
Temporal Pole	0.881	0.881
Transverse Temporal	0.683	0.843
**Parietal**		
Inferior Parietal	0.254	0.318
Postcentral	0.012*	0.06
Precuneus	0.091	0.185
Superior Parietal	0.393	0.393
Supramarginal	0.111	0.185
**Occipital**		
Cuneus	0.009**	0.02*
Lateral Occipital	0.01*	0.02*
Lingual	0.064	0.085
Pericalcarine	0.69	0.69
**Cingulate**		
Caudal Anterior Cingulate	0.868	0.868
Isthmus Cingulate	0.752	0.868
Posterior Cingulate	0.263	0.526
Rostral Anterior Cingulate	0.071	0.284

Supplementary Table 4. Comparison of p values and adjusted p values of LS Mean T1w/T2w ratio changes from baseline for a 6-month period. Raw p values and adjusted p values, using Benjamini-Hochberg procedure (FDR), are shown. For FDR, an assumption of independence between subregions within a lobe is made. Two subregions that have p values less than 0.05 have FDR Corrected p values greater than 0.05. Light orange shaded areas for $p<0.01$, light green shaded areas for $0.01\leq p<0.05$ and light-yellow shaded areas for $0.05\leq p<0.1$.

Lobe	p	FDR Corrected p
**Frontal**		
Caudal Middle Frontal	0.053	0.081
Frontal Pole	0.211	0.211
Lateral Orbitofrontal	0.211	0.211
Medial Orbitofrontal	0.059	0.081
Paracentral	0.014*	0.044*
Pars Opercularis	0.122	0.149
Pars Orbitalis	0.044*	0.081
Pars Triangularis	0.004**	0.044*
Precentral	0.016*	0.044*
Rostral Middle Frontal	0.014*	0.044*
Superior Frontal	0.058	0.081
**Temporal**		
Bankssts	0.277	0.312
Entorhinal	0.003**	0.027*
Fusiform	0.018*	0.081
Inferior Temporal	0.104	0.187
Middle Temporal	0.23	0.296
Parahippocampal	0.178	0.267
Superior Temporal	0.071	0.184
Temporal Pole	0.082	0.184
Transverse Temporal	0.66	0.66
**Parietal**		
Inferior Parietal	0.024*	0.03*
Postcentral	0.005**	0.025*
Precuneus	0.023*	0.03*
Superior Parietal	0.067	0.067
Supramarginal	0.014*	0.03*
**Occipital**		
Cuneus	0.023*	0.04*
Lateral Occipital	0.008**	0.032*
Lingual	0.03*	0.04*
Pericalcarine	0.287	0.287
**Cingulate**		
Caudal Anterior Cingulate	0.186	0.186
Isthmus Cingulate	0.184	0.186
Posterior Cingulate	0.067	0.134
Rostral Anterior Cingulate	0.056	0.134

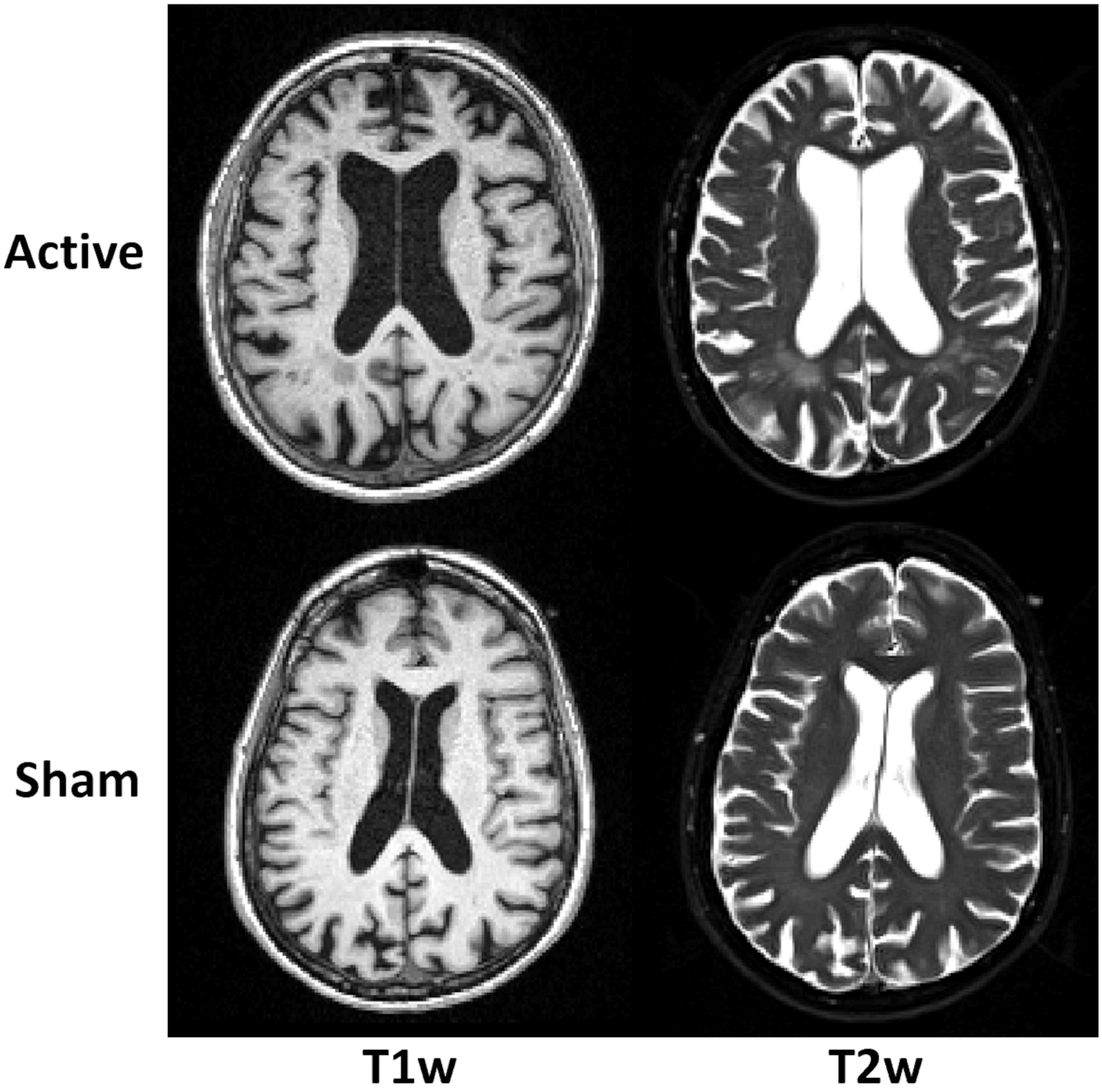

Supplementary Figure 1. T1w and T2w MRI images of a sample active treatment participant (top row) and a sham participant (bottom row). The images are shown in neurological convention.
